
JOURNAL INFORMATION
==============================
NLM Title Abbreviation: Wirtschaftsdienst
Journal ID: Wirtschaftsdienst
NLM Journal ID: 101086612

Wirtschaftsdienst (Hamburg, Germany : 1949)

ISSN: 0043-6275

ARTICLE INFORMATION
==============================
PMCID: PMC7087840
PMID: 32214513
DOI: 10.1007/s10273-020-2595-5
Article ID: 2595
Article version: 1
Subjects: Zeitgespräch

Strukturverschiebung im gesamten Abgabensystem erforderlich!

Schratzenstaller Margit margit.schratzenstaller-altzinger@wifo.ac.at

Österr. Institut für Wirtschaftsforschung, Arsenal, Objekt 20, 1030 Wien, Österreich
Electronic publication date: 2020 Mar 23
Print publication date: 2020
Volume: 100
Issue: 3
First page: 159
Last page: 161
Copyright: © Der/die Autor(en) 2020
License: Open Access Dieser Artikel wird unter der Creative Commons Namensnennung 4.0 International Lizenz veröffentlicht, welche die Nutzung, Vervielfältigung, Bearbeitung, Verbreitung und Wiedergabe in jeglichem Medium und Format erlaubt, sofern Sie den/die ursprünglichen Autor(en) und die Quelle ordnungsgemäß nennen, einen Link zur Creative Commons Lizenz beifügen und angeben, ob Änderungen vorgenommen wurden.
Die in diesem Artikel enthaltenen Bilder und sonstiges Drittmaterial unterliegen ebenfalls der genannten Creative Commons Lizenz, sofern sich aus der Abbildungslegende nichts anderes ergibt. Sofern das betreffende Material nicht unter der genannten Creative Commons Lizenz steht und die betreffende Handlung nicht nach gesetzlichen Vorschriften erlaubt ist, ist für die oben aufgeführten Weiterverwendungen des Materials die Einwilligung des jeweiligen Rechteinhabers einzuholen.
Weitere Details zur Lizenz entnehmen Sie bitte der Lizenzinformation auf http://creativecommons.org/licenses/by/4.0/deed.de.
License URL: https://creativecommons.org/licenses/by/4.0/

issue-copyright-statement: © The Author(s) 2020

==============================
Dr. Margit Schratzenstaller ist Referentin für Öffentliche Finanzen und stellvertretende Leiterin am Österreichischen Institut für Wirtschaftsforschung (WIFO) in Wien. Sie ist Expertin im Fiskalrat und Lehrbeauftragte an der Universität Wien.

Ende Februar 2020 bestätigte das Statistische Bundesamt einen Haushaltsüberschuss des Gesamtstaats nach Maastricht-Definition von 49,8 Mrd. Euro. Gegenüber dem Rekordjahr 2018, in dem Deutschland einen Maastricht-Überschuss von 62,4 Mrd. Euro erreicht hatte, ist das zwar ein Rückgang. Dennoch gab diese Meldung der anhaltenden Diskussion über die Verwendung der öffentlichen Überschüsse neue Nahrung.

Die Lage der öffentlichen Haushalte hat sich in den letzten Jahren in der Tat kontinuierlich verbessert. 2019 ist das achte Jahr in Folge, in dem der deutsche Gesamthaushalt mindestens ausgeglichen, zumeist sogar positiv bilanziert. Die Maastricht-Überschüsse stiegen seit 2014 kontinuierlich an und erreichten 2018 mit 1,9 % des Bruttoinlandsprodukts (BIP) ihren Höchststand. 2019 lag der Maastricht-Überschuss zwar unter dem Rekordstand von 2018, da unter anderem wegen der konjunkturellen Abschwächung die Ausgaben stärker stiegen als die Einnahmen. Er erreichte aber immer noch 1,4 % des BIP und übertraf damit auch die letzten Prognosen. Der um Einmalmaßnahmen und den Konjunktureffekt bereinigte strukturelle Saldo ist seit 2013 positiv und erreichte ebenfalls 2018 seinen Höchststand mit 1,4 % des BIP. Ebenso wie der Maastricht-Überschuss dürfte auch der strukturelle Überschuss 2019 zwar etwas geringer ausfallen als im Vorjahr, aber immer noch über 1 % des BIP erreichen. Mithilfe der anhaltenden Überschüsse wurde die Schuldenquote ausgehend von ihrem historischen Höchststand von 82,4 % (2010) während des vergangenen Jahrzehnts deutlich zurückgeführt: 2019 lag sie, erstmals seit 2002, wieder knapp unter 60 % des BIP, so wie in weiteren sechs alten und der Mehrheit der neuen EU-Länder.

Die wirtschaftspolitische Debatte um die „richtige“ Verwendung der öffentlichen Überschüsse wird nicht selten im Sinne eines Entweder-oder geführt: Während die eine Seite dafür plädiert, die Haushaltsspielräume primär für Investitionen zu verwenden, argumentiert die andere Seite für einen Vorrang von Steuersenkungen. Tatsächlich gibt es gute Gründe, den Haushaltsüberschuss nicht exklusiv für Steuersenkungen einerseits oder zusätzliche Investitionen andererseits zu reservieren, sondern ein Gesamtpaket von aufeinander abgestimmten Maßnahmen zu schnüren. Dieses sollte strukturelle Probleme und entsprechend finanzpolitischen Handlungsbedarf bezüglich der öffentlichen Investitionstätigkeit sowie im Abgabensystem angehen, aber auch den bestehenden konjunkturellen Risiken (internationale Handelskonflikte, Coronavirus etc.) angemessen sein.

## Ausweitung der öffentlichen Investitionen …

Dass die Finanzpolitik der letzten Jahre, die der Erhöhung nicht-investiver Ausgaben, Steuerentlastungen und dem Schuldenabbau Priorität beigemessen hat, darüber die öffentliche Investitionstätigkeit vernachlässigt hat, wie Bardt et al.[1] in der viel beachteten gemeinsamen Kurzstudie des Instituts für Makroökonomie und Konjunkturforschung (IMK) und des Instituts der deutschen Wirtschaft (IW) festhalten, ist inzwischen relativ breiter Konsens. Nachdem die Bruttoinvestitionsquote des Staates zwischen 1995 und 2007 von 2,6 % auf unter 2 % gesunken war und anschließend bei etwas über 2 % stagniert hatte, nahm sie erst in den letzten Jahren wieder leicht zu und erreichte 2019 2,5 %. Vor diesem Hintergrund beziffern Bardt et al.[2] den kumulierten Bedarf an öffentlichen Investitionen in Bildung, Forschung, Dekarbonisierung, Verkehr und Digitalisierung für das kommende Jahrzehnt auf 450 Mrd. Euro (ca. 1,3 % des BIP jährlich).

In ihrer Prognose vom vergangenen Herbst rechnet die Europäische Kommission mit einem Anstieg der Bruttoinvestitionsquote auf 2,7 % bis 2021, bestätigt aber grundsätzlich im Ende Februar 2020 veröffentlichten Länderbericht für Deutschland[3] den von IMK und IW konstatierten zusätzlichen Investitionsbedarf. Ein solches Investitionspaket würde nicht nur langfristig die Angebotsbedingungen wesentlich verbessern, sondern auch die deutsche und europäische Konjunk-

1 H. Bardt, S. Dullien, M. Hüther, K. Rietzler: Für eine solide Finanzpolitik: Investitionen ermöglichen!, IMK Report, Nr. 152, 2019.
2 Ebenda.
3 European Commission: Country Report Germany 2020, SWD(2020), 504 final, Brüssel 26.2.2020, https://ec.europa.eu/info/sites/info/files/2020-european_semester_country-report-germany_en.pdf (28.2.2020).

**Dr. Margit Schratzenstaller** ist Referentin für Öffentliche Finanzen und stellvertretende Leiterin am Österreichischen Institut für Wirtschaftsforschung (WIFO) in Wien. Sie ist Expertin im Fiskalrat und Lehrbeauftragte an der Universität Wien.

tur stabilisieren: Simulationen mit dem QUEST-Modell der Europäischen Kommission für ein an den IMK-IW-Vorschlag angelehntes Investitionspaket von 45 Mrd. Euro jährlich für die nächsten zehn Jahre legen deutlich positive Effekte für Wachstum und Beschäftigung in Deutschland sowie beträchtliche gesamtwirtschaftliche Spillover-Effekte für die Eurozone nahe.

### … und Abgabenentlastungen …

Die Haushaltsüberschüsse der vergangenen Jahre wurden wesentlich von der robusten Konjunkturentwicklung getragen, die sich in steigenden Steuer- bzw. Abgabenquoten niederschlug. So wuchs die Abgabenquote von 2012 bis 2019 um knapp 2 % des BIP auf 40,5 %, wobei sowohl die Steuerquote als auch die Sozialbeitragsquote zunahmen: die Steuerquote um 1,1 % des BIP auf 24,3 %, die Sozialbeitragsquote um 0,8 % des BIP auf 16,2 %. Die Herbstprognose der Europäischen Kommission erwartet ab 2020 entsprechend der konjunkturellen Abschwächung auch einen leichten Rückgang der Abgabenquote. Dieser ist allerdings ausschließlich durch eine sinkende Steuerquote bedingt, während die Sozialbeitragsquote stabil bleibt.

Die deutlich überdurchschnittliche Sozialbeitragsquote ist verantwortlich dafür, dass die deutsche Abgabenquote etwas über dem EU-Durchschnitt liegt (2019: 40,5 % gegenüber 39,4 % des BIP für die EU28). Die Steuerquote ist dagegen aktuell ebenso wie im langfristigen Zeitablauf unterdurchschnittlich. Entsprechend findet sich im Länderbericht der Europäischen Kommission erneut der Hinweis auf den anhaltend überdurchschnittlichen Beitrag von Steuern und Abgaben auf Arbeit zum Gesamtabgabenaufkommen, der mit knapp 57 % (2018) den EU-Durchschnitt von gut 49 % merklich überschreitet. Der Abgabenkeil (Lohnsteuer und Sozialversicherungsbeiträge) liegt in Deutschland in allen betrachteten Einkommenshöhen,[4] besonders aber im Niedrigeinkommensbereich deutlich über dem EU-Durchschnitt. Daher sollte der Fokus von Entlastungen bei den unteren und mittleren Einkommen liegen. Dabei sollten sich Entlastungsmaßnahmen nicht auf die Einkommensteuer beschränken, sondern unbedingt auch die Sozialbeiträge mit einbeziehen. So könnte etwa im Niedrigeinkommensbereich ein Grundfreibetrag für Arbeitnehmerbeiträge eingeführt werden, der mit zunehmendem Einkommen abgeschmolzen wird, um gezielt Geringverdiener zu entlasten.[5] Eine alternative Option wäre die begrenzte Anrechnung von Sozialbeiträgen bei der Einkommensteuer nach dem österreichischen Vorbild einer „Negativsteuer“. Im Bereich des Einkommensteuertarifs könnte der Grundfreibetrag erhöht werden, was allerdings im Vergleich zu den genannten Optionen für eine Senkung der Sozialbeiträge nicht gezielt Geringverdiener, sondern alle Einkommensteuerpflichtigen entlasten würde. Die Senkung der Steuerlast für die Mittelschicht könnte die Abflachung des „Mittelstandsbauchs“, also der steilen Tarifprogression im Eingangsbereich des Einkommensteuertarifs, bringen,[6] aber auch eine Erhöhung der Einkommensgrenze von derzeit 56 000 Euro jährlich, ab der der Spitzensteuersatz greift. Nicht zuletzt wären auch Optionen zur Einschränkung des Ehegattensplittings, mit seinen problematischen Wirkungen auf Verteilung und Arbeitsanreize, wieder zu diskutieren. Entlastungsmaßnahmen im Bereich von Einkommensteuer und Sozialbeiträgen, insbesondere jene für Geringverdiener, wären nicht nur strukturell zur Stärkung der Arbeitsanreize – insbesondere für Frauen, deren Arbeitsangebot besonders sensibel auf die Steuer- und Abgabenbelastung reagiert – sinnvoll, sondern könnten auch zur Konjunkturstabilisierung beitragen.

Beflügelt durch die kürzlich gesenkte Körperschaftsteuer in den USA und in Großbritannien sowie Ankündigungen künftiger Körperschaftsteuersatzsenkungen etwa in Österreich wurden in den letzten Monaten in Deutschland ebenfalls Rufe nach einer Senkung der Körperschaftsteuer laut. Der Länderbericht der Europäischen Kommission weist ebenfalls auf die relativ hohe Unternehmensteuerbelastung in Deutschland hin. Eine allgemeine Körperschaftsteuersenkung wäre allerdings nicht nur mit relativ hohen Steuerausfällen verbunden, sondern auch wenig zielgerichtet. Stattdessen sollten gezielte Anreize im Unternehmensteuersystem gesetzt werden, indem etwa die Abschreibungsbedingungen oder die steuerliche Forschungsförderung verbessert werden: Eine solche zielgerichtete steuerliche Förderung von privaten Investitionen, die den Großteil der Gesamtinvestitionen ausmachen, in die Modernisierung der Volkswirtschaft wäre eine zielführende Ergänzung eines öffentlichen Investitionspakets.[7]

### … und wie sich beides vereinbaren lässt

Die Europäische Kommission erwartet für 2020 und 2021 einen weiteren Rückgang der Schuldenquote, auf 56,8 % bzw. 55 % des BIP, ebenso wie weitere Haushaltsüberschüsse. Somit wäre auch in diesem und im kommenden Jahr ein gewisser Budgetspielraum vorhanden. Es ist aber absehbar, dass er infolge der schwächeren Konjunktur sowie bereits

4 Zwischen 50 % und 167 % eines Durchschnittseinkommens.

5 Vgl. hierzu und zu den folgenden Entlastungsoptionen die Mikrosimulationen von S. Bach, P. Haan, M. Harnisch: Progressive Sozialbeiträge können niedrige Einkommen entlasten, in: DIW-Wochenbericht, 13+14/2018, S. 278-285.

6 Vgl. S. Bach: Wie sich der Soli abschaffen ließe, ohne die Ungleichheit zu erhöhen, Makronom Blog, 28. 8.2018, https://makronom.de/wie-sich-der-soli-abschaffen-liesse-ohne-die-ungleichheit-zu-erhoehen-27586 (28.2.2020).

7 J. Südekum: Investitionen brauchen Steuerung, in: Tagesspiegel, 17.1.2020, https://causa.tagesspiegel.de/politik/ist-jetzt-der-richtige-zeitpunkt-fuer-steuersenkungen/investitionen-brauchen-steuerung.html (28.2.2020).

geplanter zusätzlicher Ausgaben (wie die Grundrente) bzw. Steuersenkungen (teilweise Abschaffung des Solidaritätszuschlags spätestens ab 2021), die auch weiterhin für ein schnelleres Wachstum der Ausgaben gegenüber den Einnahmen sorgen, allmählich schrumpft.[8] Die Europäische Kommission geht für 2020 und 2021 von einem rückläufigen Maastricht-Überschuss von 0,6 % bzw. 0,2 % und von einem ebenfalls sinkenden strukturellen Überschuss von 0,7 % bzw. 0,5 % aus.[9] Gemessen an der Differenz zwischen strukturellem Überschuss und mittelfristigem Haushaltsziel von einem strukturellen Defizit von maximal -0,5 % des BIP beläuft sich somit der prognostizierte fiskalische Spielraum 2020 auf 1,2 % und 2021 auf 1 % des BIP. Dessen Nutzung für die Ausweitung öffentlicher Investitionen hat nicht nur die Europäische Kommission, sondern auch der Internationale Währungsfonds wiederholt empfohlen.

Die zu erwartenden Haushaltsüberschüsse sowie die Ausgabenspielräume, die Schuldenbremse und EU-Fiskalregeln ermöglichen, werden den Investitionsbedarf der kommenden Jahre allerdings nur teilweise decken können. Möglichkeiten zur Modifikation des geltenden fiskalischen Regelwerks – etwa im Sinne einer „Goldenen Regel“, die den Verschuldungsspielraum für Nettoinvestitionen erweitert – diskutiert der IMK-IW-Vorschlag für ein öffentliches Investitionspaket. Interessant ist auch das Konzept einer „grünen goldenen Investitionsregel“[10], die erweiterte Verschuldungsspielräume für „grüne“ öffentliche Investitionen vorsieht.

Jedenfalls würden die Umsetzung eines umfangreichen Investitionspakets, spürbare Steuer- und Abgabensenkungen für kleinere und mittlere Einkommen sowie gezielte steuerliche Investitionsanreize für Unternehmen den absehbaren Budgetspielraum übersteigen. Es gilt zu bedenken, dass aufgrund der bereits beschlossenen Teilabschaffung des Solidaritätszuschlags mit einem jährlichen Entlastungsvolumen von über 10 Mrd. Euro spätestens ab 2021 und der konjunkturellen Abschwächung die Steuer- und Abgabenquote nach ihrem Höchststand 2019 ohnehin allmählich wieder leicht zurückgehen werden.[11]

Die verkürzte steuerpolitische Sicht auf die leicht überdurchschnittliche Abgabenquote, die durch Steuerentlastungen zurückgeführt werden müsse, verstellt den Blick auf strukturellen Reformbedarf im deutschen Abgabensystem.[12] Der Spielraum für konjunkturell wie strukturell sinnvolle Abgabenentlastungen für untere und mittlere Einkommen sowie steuerliche Investitionsanreize für Unternehmen kann erweitert werden, indem diese in eine ebenfalls seit längerem von den internationalen Organisationen empfohlene Strukturverschiebung im gesamten Abgabensystem eingebettet werden. So hat das Gewicht umweltbezogener Abgaben in Deutschland seit Mitte der 2000er Jahre deutlich abgenommen. Bezogen auf das BIP gingen sie 2018 auf 1,8 % zurück, ihr Anteil an den Gesamteinnahmen durch Abgaben fiel auf 4,5 %; damit befindet sich Deutschland auf einem der hintersten Ränge in der EU.[13] Der ab 2021 geplante Einstieg in eine $CO_2$-Bepreisung wird zumindest kurzfristig an diesem Ungleichgewicht wenig ändern. Auch werden die Einnahmenpotenziale vermögensbezogener Steuern zu wenig ausgeschöpft: Die Grundsteuer trägt mit 1,1 %, die Erbschaftsteuer mit 0,4 % zu den gesamten Einnahmen durch Abgaben bei.

Die stärkere Nutzung umwelt- und vermögensbezogener Steuern würde Mehreinnahmen erbringen, die die angesprochenen Entlastungen bei Einkommensteuer und Sozialbeiträgen finanzieren helfen. So sollten die Basis bei Umweltsteuern verbreitert und umweltkontraproduktive Steuerausnahmen eingeschränkt werden. Eine Reform der Erbschaftsteuer, die eine deutliche Verbreiterung der Bemessungsgrundlage (insbesondere im Bereich der Besteuerung von Unternehmensübertragungen) mit geringen Steuersätzen kombiniert, könnte insgesamt höhere Einnahmen erbringen. Mit der jüngsten Reform der Grundsteuer wurde durch die aufkommensneutrale Ausgestaltung die Chance vergeben, die Grundsteuereinnahmen insgesamt zu erhöhen; hier könnte über entsprechende Anpassungen nachgedacht werden.

Eine Verschiebung der Abgabenlast weg von Arbeit hin zu Umweltverbrauch und Emissionen sowie Grundvermögen und Erbschaften würde das Abgabensystem wachstums- und beschäftigungsfreundlicher, sozial inklusiver und ökologisch nachhaltiger machen. Zusammen mit einer groß angelegten, teilweise defizitfinanzierten öffentlichen Investitionsoffensive würde eine derartige Abgabenstrukturreform einen wichtigen Beitrag zur Bewältigung der bestehenden Herausforderungen – Klimawandel und Energiewende, Digitalisierung, demografischer Wandel, Defizite bei der Gleichstellung von Frauen und Männern im Erwerbsleben – leisten.

8 C. Michelsen, M. Clemens, M. Hanisch, S. Junker, L. Pagenhardt, T. Schlaak: Deutsche Industrie: Wirtschaft kämpft sich mühsam aus der Krise, in: DIW-Wochenbericht, 50/2019, S. 936-950.

9 European Commission, a. a. O.

10 Vgl. G. Claeys, S. Tagliapietra, G. Zachmann: How to Make the European Green Deal Work, Bruegel Policy Contribution, Nr. 13, 5.11.2019, https://www.bruegel.org/2019/11/how-to-make-the-european-green-deal-work/ (28.2.2020).

11 K. Rietzler: Steuersenkungen sind eine Gefahr, in: Tagesspiegel, 17.1.2020, https://causa.tagesspiegel.de/politik/ist-jetzt-der-richtige-zeitpunkt-fuer-steuersenkungen/steuersenkungen-sind-eine-gefahr.html (28.2.2020).

12 Vgl. dazu auch M. Schratzenstaller: Ökosoziale Abgabenstrukturreform, in: Wirtschaftsdienst, 97. Jg. (2017), H. 6, S. 400-403, https://www.wirtschaftsdienst.eu/inhalt/jahr/2017/heft/6/beitrag/wie-sollte-das-steuersystem-in-deutschland-reformiert-werden.html (28.2.2020).

13 European Commission, a. a. O.
